# Does micro-granulated yeast probiotic (*Saccharomyces cerevisiae*) supplementation in milk replacer affect health, growth, feed efficiency and economic gain of calves?

**DOI:** 10.1016/j.vas.2023.100329

**Published:** 2023-12-23

**Authors:** Nizar Salah, Héloïse Legendre, Valentin Nenov, Maxime Briche, Flore Serieys, Silvia Grossi, Carlo Angelo Sgoifo Rossi

**Affiliations:** a Phileo by Lesaffre, 137 rue Gabriel Péri, 59700 Marcq-en-Baroeul, France; b l'INP ENSAT Avenue de l'Agrobiopole, 31326 Auzeville-Tolosane, France; c University of Milan, Department of Veterinary Science for Health, Animal Production and Food Safety, Via Dell'Università 1, 26900 Lodi, France

**Keywords:** Yeast probiotic, Calves, Growth, Efficiency, Health

## Abstract

The goal of calf feeding systems is to provide calves with optimum nutrition to promote growth, health, and future milk production and to reduce antibiotic use which leads to a need for alternatives that reduce illness and promote growth in dairy calves. We hypothesized that feeding live yeast would improve gastrointestinal health and growth performance of calves. The aim of this study was then to evaluate the effects of supplementing a yeast probiotic *Saccharomyces cerevisiae* (CNCM I-4407, 10^10^ CFU/g, Actisaf® Sc47 powder; Phileo by Lesaffre, France) in milk replacers (MR), on health and growth of pre-weaned calves. Forty Holstein female calves were used during this trial. Each calf was weighed at 3 days of age and then introduced in the trial. Calves were randomly assigned to 2 groups (*n* = 20/group) and were fed MR without (control; CON) or with yeast probiotic at 1 g/calf/d (experimental; EXP). Milk replacer (12.5 % solids) was offered twice a day until 66 days of age (DOA). Body Weight (BW), wither height, hip width, body length and chest girth were collected in day 3 and day 66. Compared to CON, calves supplemented with yeast probiotic had better average daily gain (ADG, 0.456 ± 0.1 *vs.* 0.556 ± 0.09 kg/d, *p* < 0.05). There was no difference (*p >*  0.05) in both starter and MR intake between the two groups. Feed efficiency was better (*p* < 0.05) in the EXP group (2.18 ± 0.53) compared to CON (2.63 ± 0.78). No statistical differences were found between groups even if the lower total morbidity (40.91 % in the CON vs*.* 19.05 % in EXP) and incidence of gastrointestinal disorders (36.36 % in the CON vs. 14.29 % in EXP) were observed in calves supplemented with yeast probiotic. The severity of diarrhea was numerically lower in calves supplemented with yeast probiotic. No severe cases of respiratory disorders were highlighted in the present trial. The cost/kg of gain was higher (*p <*  0.05) in CON compared to EXP group. Total expenses linked to feeds and veterinary treatments were higher in CON compared to EXP group. During the study, the use 1 g/d of yeast probiotic allows to save 32.86 €/calf.

It could be concluded that supplementing Actisaf® powder (Actisaf® SC 47 PWD) in MR improved health, growth performance, feed efficiency, and reduced the expenses linked to feeds and veterinary treatments.

## 1 Introduction

Several studies have shown the effect of early life health and growth of calves on future production (Geslinger et al., 2016; Soberon & Van Amburgh, 2013). Efficiently rising female replacement calves is a crucial point to improve the overall production of the herd and the farm profitability, as well as to reduce the use of antibiotics, to counteract the problem of antimicrobial resistance (AMR) and to adhere to the new European standards (Cangiano et al., 2020; WHO, 2019). Since the ban on the use of certain antibiotics as growth promoters in animal feed due to the apparition of antibiotic resistance, the need to find alternatives has accelerated to reduce antibiotic use worldwide and particularly in countries with the highest antibiotic consumption such as Italy (European Medicines Agency, 2019).

To reduce antibiotics, probiotics such as live yeast have been considerably used over the last 15 years especially in mature ruminants and monogastrics fed solid feeds compared to pre-weaned calves fed milk replacers (Chaucheyras-Durand & Durand, 2010; Davies et al., 2022). Even if the results in terms of growth performance in pre-weaned calves are extremely variable, with either positive (Hassan et al., 2016; Saldana et al., 2019) or no effect (Galvão et al., 2005; He et al., 2017), there is a strong evidence that the administration of live yeast in the preweaning period positively affects the health status (Cangiano et al., 2020; He et al., 2017; Zhang et al., 2021) and indirectly increase growth performance. Reported as the primary source of calf mortality during the pre-weaning period (Cho & Jin Yoon, 2014), the incidence, severity and duration of diarrhea was significantly reduced by the inclusion of yeast mainly by improving the development of the intestinal epithelium, mucosa and local immunity, and by preventing pathogenic bacteria from binding to intestinal epithelial cells (Villot et al., 2020; Magalhães et al., 2008; Galvão et al., 2005). The results can be influenced by the route of administration and the dose. The inclusion of yeast in solid feed is more correlated with rumen development, metabolism, and an increase growth rate, while its inclusion in the MR is more correlated to a better intestinal health and reduced diarrhea incidence (Alugongo et al., 2016; Cangiano et al., 2020). To our knowledge, there are few data testing the effect of live yeast in MR on growth performance, efficiency, and health status of calves during the pre-weaning period.

Therefore, the objective of this study was to evaluate the effects of supplementing micro-granulated, heat stable yeast probiotic special form of Actisaf® Sc47 powder **(PWD)** in milk replacer during the pre-weaning period on growth, feed efficiency and health of calves.

## 2 Materials and methods

### 2.1 Animals and experimental design

The experimental trial was done in line with the Italian requirements on animal welfare, according to the Legislative Decree n° 126/2011 on calf management. No experimental practices that will or can harm the animals or put their welfare at risk were done.

The study was performed in an Italian commercial dairy farm (Azienda Agricola Del Santo, Castelgerundo, 26,823 LO) with more than 300 lactating cows and high-quality management level. The study started on the 20th of February 2022, when the first calf enrolled was born, and finished on the 31st of August 2022, when the last enrolled calves were weaned at 66 days of age. Each calf remained in the trial from 3 days of age to 66 days, and because all calves were not introduced in the trial at the same time, the trial lasted 180 days.

The study included 40 female Holstein–Friesian calves from birth to weaning. Within 6 h after calving, calves were removed from their dam and received 4 L/d of good quality colostrum (≥ 22 % Brix) for the first 3 days. Animals were housed in individual calf boxes (2.0 × 1.55 m) with straw bedding and fed two times daily (at 8 a.m and 5 p.m). Calves had ad libitum access to water and were provided with hay and calf starter concentrate. Each calf was weighed at 3 days of age and then introduced in the trial to obtain at the end 2 groups of 20 calves each. Calves in the control (CON) group were fed a starter concentrate and MR without any yeast probiotic supplementation (*n* = 20, initial body weight = 40.60 ± 3.83 kg). Calves in the experimental group (EXP, *n* = 20, initial body weight = 40.75 ± 3.07) were offered the same starter concentrate and MR as the CON group and supplemented with 1 g/calf/day of yeast probiotic. Yeast probiotic was used in micro-granulated (Actisaf® Sc 47 PWD) form and supplemented via MR. Animals of the two groups received the same quantity of MR (same volume and concentration: 125 g/L of meal) according to the following program (Table 1) and were fed MR in open buckets. The nutritional characteristics of MR and concentrate are given in Table 2.

**Table 1:** Milk feeding program during the trial.

Age	Meal/day	Quantity/meal (L)
Day 1–3	2–3	colostrum
Day 4–7	2	2
Week 2	2	2.5
Week 3	2	3
Week 4	2	3.5
Week 5	2	3.5
Week 6	2	3.5
Week 7	2	3
Week 8	1	2
Week 9	1	2
Week 10	0	weaning

**Table 2:** Ingredients and chemical composition of experimental diets.

Item	Calf starter	Milk replacer
Ingredients (%)		
Rolled corn meal	34	
Dry beet pulp	18	
Soybean meal 48 CP	13.71	
Rolled barley	12	
Wheat bran	10	
Molasses cane	5	
Soybean meal extruded	5	
Mineral and vitamin mix	2	
Slow-releasing urea	0.25	
Flavor	0.04	
Chemical composition (% DM)		
Crude protein	17.20 %	22.8 %
Crude fat	3.50 %	21.40 %
Crude fiber	8.40 %	0.03
Moisture	11.00	3.20 %
Ash	7.30 %	7.12 %
Calcium	0.84 %	0.81 %
Phosphorus	0.41 %	0.63 %
Sodium	0.2 %	0.7 %

### 2.2 Growth performance and feed efficiency

For every calf, the date of birth was registered. The body weight (**BW**), wither height, chest girth, hip width and body length were measured at the end of the colostrum administration phase (3 days after calving) and at weaning (66 days). Individual solid feed and milk replace intake was recorded daily from the beginning to weaning. Feed efficiency was calculated by dividing total dry mater intake by body weight gain.

### 2.3 Health status

Mortality, morbidity, pathological disorders, and veterinary treatments were recorded. For diarrhea, we used fecal score as parameter according to the method described by Magalhães et al. (2008). Briefly, fecal score was recorded as 1 when firm, 2 when soft or of moderate consistency, 3 when runny or as mild diarrhea, and 4 when watery and pro-fuse diarrhea. The potential diarrhea was defined when the fecal score was 2, and the diarrhea was defined when the fecal score was above 2. Detection of diarrhea was visual during an individual check every day and no follow-up was done overnight. Fecal score was not categorized as number of days with fecal score ≥ 3.

For respiratory disorder we used tow criteria based on Wisconsin health scoring gird as described by Vandermeulen et al. (2016). Briefly, respiration disorder was recorded according to coughing frequency as 1 for induce single cough, 2 for induced repeated coughs or occasional spontaneous coughs and 3 for repeated spontaneous cough. We used also nasal discharge and, respiratory disorder was recorded as 1 for small amount of unilateral cloudy discharge, 2 for bilateral, cloudy or excessive mucus discharge and 3 for copious bilateral mucopurulent discharge. The respiratory disorder was defined when the coughing frequency was 2 or above and when nasal discharge score was 2 or above. Detection of respiratory was visual during an individual check every day and no follow-up was done overnight. Nasal discharge and coughing frequency were verified twice a day for each calf.

### 2.4 Economic analysis

Economic analysis was performed according to starter intake and price, MR intake and price and the different veterinary treatments against diarrhea and respiratory diseases. The input values in the calculation were as follows: 500€/T of starter; 2500€/T of MR. Labor cost was not computed. For veterinary treatments, cost was considered based on market price and the dose for each product used to treat diarrhea (Gentamicin: 0.64€, Mineral: 0.65€ and sodium bicarbonate/glucose: 4€) and respiratory diseases (Amoxicillin/clavulanic acid: 1.4€; Meloxicam: 0.56€). The difference between the two groups indicated the economic gain linked to feed and veterinary treatment expenses. Total cost linked to veterinary treatments and feeds is calculated as follow.

$$Cost\;of\;diarrhea=N^{\circ }\;\text{of}\;\text{calves}\;\text{with}\;\text{diarrhea}\;x\;\text{cost}\;\text{of}\;\text{each}\;\text{treatment}$$

$$Cost\;of\;respiratory\;disorders\;\left(RD\right)=N^{\circ }\;\text{of}\;\text{calves}\;\text{with}\;\text{RD}\;x\;\text{cost}\;\text{of}\;\text{each}\;\text{treatment}$$

Totalcostofveterinarytreatments(allsickcalves)=Costofdiarrhea+costofRD

$$Cost\;of\;veterinary\;treatments\;per\;calf=\frac{Total\;cost}{Number\;of\;sick\;calves}$$

Savingslinkedtoveterinarytreatments=costCONgroup−costEXPgroup

Totalcostoffeeds=(Intakeofsolidfeedxprice)+(IntakeofMRxprice)

$$\frac{Cost}{Kg\;of\;gain}=\frac{Total\;cost\;of\;feeds}{Body\;weight\;gain}$$

Savingslinkedtofeeds=costCONgroup−costEXPgroup

Totalsavings=Savingslinkedtoveterinarytreatments+savingslinkedtofeeds

### 2.5 Statistical analysis

**Fig. 1:**
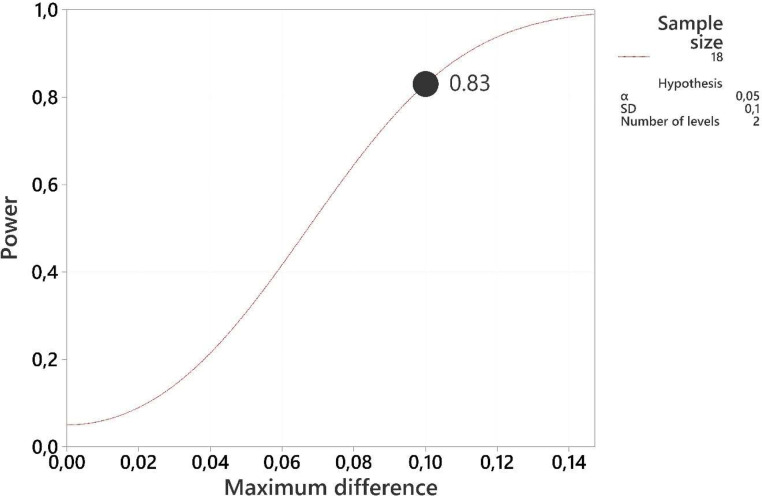
Power curve to estimate sample size according to BWG.

For the primary response variables, including BWG and ADG a power analysis was conducted to estimate sample size (Hintze, 2008; Morris, 1999) based on previously published values (Miller-Cushon & DeVries, 2011). From the power test analysis with α = 0.05 and power = 0.80, the predicted sample size was 18 and 17 calves per treatment for BWG and ADG (Fig. 1). Our design does not have enough power according to DM intake.

The data of the experiment were tested for normality using Shapiro-Wilk test, and were found normally distributed, then were statistically analyzed using mixed effect model procedure of (Minitab® 15.1.30.0., 2007). Calf was the experimental unit as they were housed individually, and measurements were taken and known for each animal. The following model was used to analyze growth performance, feed efficiency and morphometric measurements.

$$Y_{\text{ijkl}}=\mu +T_{i}+A_{j}+B_{k}+C_{l}+e_{\text{ijkl}}$$

Where Y_ijkl_ is the explained variable, µ: general mean, T_i_: treatment effect (with vs. without yeast probiotic), A_j_ is fixed effect month of birth, B_k_ is the initial body weight used as covariate, C_l_: the random effect of the calf, and eijkl is the residual effect. The results were reported as least square means and standard error of means (SEM). Unstructured covariance which had the lowest Akaike information criterion (AIC), was used. For each variable, a graphic verification of data quality was done via boxplot to identify outliers. Outliers were considered for data appearing with an asterisk in the boxplot (and thus for values with ±1.5 interquartile range) and outside the 95 % confidence interval of the normality probability plot.

The mortality, frequency of diseases, the incidence and severity of diarrhea and respiratory disease were assessed by applying a chi-squared test (PROC FREQ).

For all parameters, the level of statistical significance was set at *p <*  0.05. A tendency for significance was declared at 0.05 ≤ *p <*  0.10.

## 3 Results

### 3.1 Growth performance, feed intake and efficiency

Table 3 reports the body weight at weaning (**BWW**), body weight gain (**BWG**), average daily gain (**ADG**), intake, and feed efficiency (FE). Initial BW, MR and starter DMI were not significantly different between treatments (*p >*  0.1). However, BWW, BWG and ADG were significantly (*p* = 0.006) higher for calves supplemented with Actisaf® Sc 47 PWD than for control calves. Better FE was observed in EXP group (2.18) compared to CON (2.63). Calves supplemented with Actisaf® Sc 47 PWD required significantly less feed per kg of BW gain than control (*p* = 0.04).

**Table 3:** Effect of yeast probiotic Actisaf® *Sc* 47 PWD in milk replacer on growth, feed intake and efficiency of calves.

Item	Treatment^2^			
	CON	EXP	SEM	*P*-value
BW at d 3, kg	40.60^a^	40.80^a^	0.88	0.90
BWW at d 66, kg	69.41^b^	75.71^a^	1.25	0.006
BWG, kg	28.47^b^	35.04^a^	1.25	0.006
ADG, kg/d	0.456^b^	0.556^a^	0.03	0.006
Total MR intake, kg of DM	43.14^a^	43.89^a^	0.37	0.11
Total starter intake^3^, kg of DM	28.37^b^	30.08^a^	1.3	0.25
FE^1^	2.63^b^	2.18^a^	0.20	0.04

a-b Mean values in the same row with different superscripts differ significantly (*P* < 0.05).

1 FE = feed conversion ratio (DM intake/body weight gain).

2 CON = milk replacer without Actisaf® Sc47 PWD; EXP = milk replacer with 1 g/d of Actisaf® Sc47 PWD.

Body measures at birth (d 3) and at weaning (d 66), in terms of centimeters of wither height, hip width, chest girth and body length are reported in Table 4. The inclusion of Actisaf® Sc 47 PWD significantly (*P* < 0.05) improved the wither height gain (11.46 *vs.* 13.81 cm for CON and EXP groups, respectively) and body length gain (12.26 *vs.* 14.72 cm for CON and EXP groups, respectively). There was no effect of Actisaf® Sc 47 PWD on hip width and chest girth gain (*p >*  0.1).

**Table 4:** Effect of yeast probiotic Actisaf® Sc 47 PWD in milk replacer on body measurements of calves.

Item	Treatment^2^			
	CON	EXP	SEM	*P*-value
Wither height at d 3, cm	72.4^a^	72.5^a^	0.83	0.99
Wither height at d 66, cm	83.96^a^	86.31^b^	0.48	0.02
Wither height gain, cm	11.46^a^	13.81^b^	0.48	0.02
Body length at d 3, cm	61.0^a^	61.1^a^	0.84	0.93
Body length at d 66, cm	73.31^a^	75.77^b^	0.56	0.04
Body length gain, cm	12.26^a^	14.72^b^	0.56	0.04
Hip width at d 3, cm	20.65^a^	20.6^a^	0.24	0.88
Hip width at d 66, cm	23.67^a^	24.24^a^	0.18	0.14
Hip width gain, cm	3.04^a^	3.62^a^	0.18	0.14
Chest girth at d 3, cm	79.85^a^	79.65^a^	0.77	0.85
Chest girth at d 66, cm	101.7^a^	103.64^a^	0.59	0.11
Chest girth gain, cm	21.95^a^	23.88^a^	0.59	0.11

a-b Mean values in the same row with different superscripts differ significantly (*P* < 0.05).

2 CON = milk replacer without Actisaf® Sc47 PWD; EXP = milk replacer with 1 g/d of Actisaf® Sc47 PWD.

### 3.2 Health status

During the trial, 3 calves died because of diarrhea, 2 in the CON group, and 1 in the EXP group. The dead calves were replaced to maintain the same number of calves in each group. Total morbidity and incidence of the different diseases are reported in Table 5. No statistical differences were found between groups even the lower total morbidity (40.91 % in the CON vs. 19.05 % in EXP) and incidence of gastrointestinal disorders (36.36 % in the Control vs. 14.29 % in EXP) observed in calves supplemented with Actisaf® Sc47 PWD. Also, the severity of the cases of diarrhea appeared lower in calves supplemented with Actisaf® Sc47 PWD but the differences did not reach the statistical significance. No severe cases of respiratory disease were highlighted in the present trial. During the trial, a total number of 8 and 3 calves received veterinary treatments to treat diarrhea from CON and EXP, respectively.

**Table 5:** Health status of calves during the trial.

Item		Treatment 1	
	Control	EXP	*P*-value
N° of Calves	20	20	
Mortality (%), n	10 %, (2)	5 %, (1)	NS
Morbidity (%), n	40,91 %, (9)	19,05 %, (4)	NS
Incidence of (%), n			
Respiratory disorders	4,55 %, (1)	4,76 %, (1)	NS
Diarrhea	36,36 %, (8)	14,29 %, (3)	NS
Diarrhea severity,% of total score, n			
2	50 %, (4)	66,67 %, (2)	NS
3	50 %, (4)	33,33 % (1)	NS

1 CON = milk replacer without Actisaf® Sc47 PWD; EXP = milk replacer with 1 g/d of Actisaf® Sc47 PWD NS = non-significant.

### 3.3 Economic gain

**Fig. 2:**
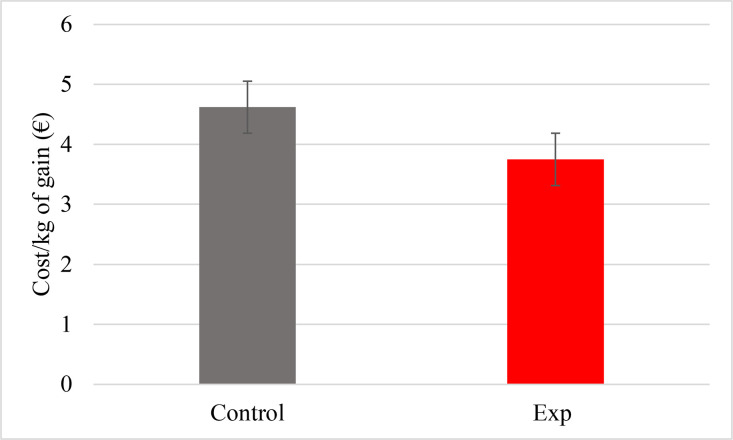
Effect of Actisaf® Sc47 PWD on economic gain. EXP = milk replacer with 1 g/d of Actisaf® Sc47 PWD. (For interpretation of the references to color in this figure legend, the reader is referred to the web version of this article.)

The effect of Actisaf® Sc47 PWD on economic gain linked to the feed is presented in Fig. 2. The supplementation of Milk replacer by 1 g/d of Actisaf® Sc47 PWD reduced significantly (*p* = 0.038) the cost/kg of gain by 17.8 % (4.53 vs. 3.72 € for CON and EXP groups, respectively). For a calf that gains 40 kg of BW, the cost is 181.2 € for CON group and 148.8 € for EXP group which corresponds to a save of 32.4 €.

**Fig. 3:**
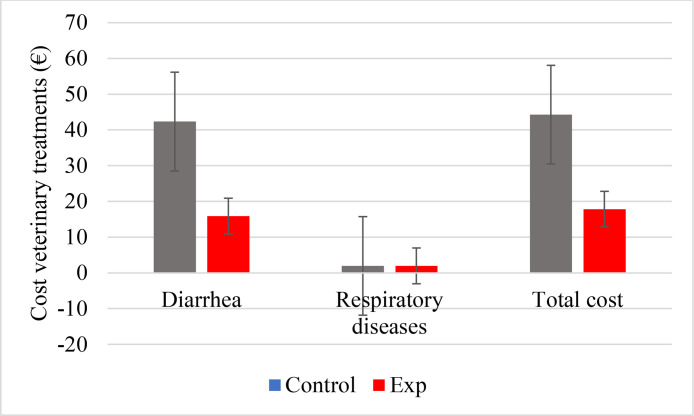
Effect of Actisaf® Sc47 PWD on economic gain linked to veterinary treatments during the trial for all sick calves. EXP = milk replacer with 1 g/d of Actisaf® Sc47 PWD. (For interpretation of the references to color in this figure legend, the reader is referred to the web version of this article.)

The effect of Actisaf® Sc47 PWD on economic gain linked to veterinary treatments for all sick calves is presented in Fig. 3. The use 1 g/d of Actisaf® Sc47 PWD reduced the cost of products used to treat diarrhea by 62.5 % (42.32 vs. 15.87 € for Control and EXP groups, respectively). There was no effect of Actisaf® Sc47 PWD on expenses related to respiratory diseases which were around €1.96 for the two groups. The total cost of veterinary treatments was higher in control group (44.28 € for all sick calves) compared to EXP group (17.83€ for all sick calves). In total, nine calves were sick in CON group (8 with diarrhea and 1 with respiratory diseases) and four were sick in EXP group (3 with diarrhea and 1 with respiratory diseases) which corresponds to a cost of 4.92 and 4.62 €/calf for CON and EXP groups, respectively. The use of 1 g/d of Actisaf® Sc47 PWD in milk replacer allows to save 0.46€/calf. The total savings related to veterinary treatments and feeds is 32.86 in EXP group.

## 4 Discussion

Globally, the use of probiotics as an alternative to antibiotics in agriculture practice has increased because of the increasing concerns around antibiotic resistance (Hume, 2011). A variety of microbial species, such as Bacillus spp., Enterococcus spp. and Saccharomyces yeast (Simon et al., 2001), have been used in livestock as probiotics products. The present study investigated the effects of supplementing a live yeast on dairy calf growth performance and its potential to improve animal health.

In the current study, Actisaf® Sc47 PWD supplementation increased growth performance and feed efficiency. These results are consistent with those presented by Galvão et al. (2005) who showed an ameliorative effect of 0.5 g of live yeast *S.cerevisiae var. boulardii* on growth performance (+ 39 % ADG) and feed efficiency (+ 19 %) of calves prior to weaning. Lee et al. (2019) studied the effect of 3 doses of live yeast (0.5, 1 and 2 g/d) incorporated into MR during normal and heat stress conditions. During the two periods, they observed higher ADG of calves supplemented with 1 g of live yeast than the control and the two other doses although the difference was not significant. Also, calves supplemented with 1 g were more sufficient during thermal neutral conditions. Similarly, Davies et al. (2021) compared 4 groups of calves according to the presence or absence of nutritional stress and the inclusion or not of yeast at 1 g/d in MR. They observed a trend towards a positive effect of live yeast on growth performance and indicated that this effect is greater under nutritional stress conditions. In agreement, Siachos et al. (2022) observed improvements in ADG (+ 19 %) and feed efficiency when fed live yeast strain *S.cerevisiae var. boulardii CNCM I-1079* incorporated into MR at 5 g/d which corresponds to 2 × 10^9^ CFU/d.

During our research, we found more studies on yeast culture than live yeast and more data on the use of yeast in solid feed than milk replacers. Zhang et al. (2021) carried out a meta-analysis containing 34 studies and 2126 calves among them 13 were supplemented with live yeast and 21 were supplemented with yeast fermentation products. They observed positive effect of *saccharomyces cerevisiae* products on ADG and dry matter intake among preweaning calves, but not among postweaning calves. Separating the live yeasts and yeast fermentation products, they observed a tendency for higher ADG with live yeast and no effect of fermentation yeast products. Discrepancies in results might be attributed to calf management, microbial properties including stain, type of product, dosage, form of and route of delivery: milk or starter (Cangiano et al., 2020; Davies et al., 2022.). The effect of live yeast has been well studied in adult ruminants either in vitro or in vivo. In calves, the same effects can be observed if the supplementation is done via solid feed which directly impact the rumen environment (Hassan et al., 2016). Within the rumen, live yeasts optimize rumen environment which stimulate microbial growth especially fibrolytic populations, fiber digestion, nutrient availability, and consequently better growth performance (Villot et al., 2022; Zhang et al., 2021).

In our study, yeast probiotic Actisaf® Sc47 PWD was provided via the MR and consequently passed directly from the esophagus to the abomasum, resulting in little or no ruminal effects (Hill et al., 2008). It has been demonstrated that in veal calves fed MR, 3 % of milk can enter the rumen without any problem. We can hypothesize that during our trial some of milk entered the rumen and the Actisaf® Sc47 PWD is incorporated into the rumen and thus modulate ruminal fermentation with some effects on calf growth (Hassan et al., 2016). The higher growth performance and feed efficiency of calves supplemented with Actisaf® Sc47 PWD can be explained by better health status.

During our trial, more sick calves were observed in CON group compared to EXP group. It is thus possible that these calves may use more energy to maintain their basal metabolism and recovery and less energy for growth leading to their lower performances (Gebremedhin et al., 1981; Orellana et al., 2019). Xiao et al. (2016) demonstrated that supplementation of calves with *Saccharomyces cerevisiae* products increased villus height-to-crypt depth ratio of intestinal papillae and consequently better absorption, growth, and efficiency. Similar results were also found in piglets (Shen et al., 2009) and broilers (Gao et al., 2008).

No direct measurement of intestinal or gut health was followed during our study, but both diarrhea and respiratory diseases were monitored. Diarrhea and respiratory disorders are responsible for most of the calf mortality and morbidity especially during the preweaning period (McGuirk, 2008; Smith, 2013; Urie et al., 2018). Supplementation of Actisaf® Sc47 PWD in MR reduced the number of calves with diarrhea, the incidence and severity of diarrhea but had no effect on the proportion of calves affected by respiratory disorder. It has been shown that live yeast reduce diarrhea by preventing pathogenic bacteria from binding to intestinal epithelial cells, by modulating gut mucosal immunity or increased relative abundance of beneficial bacteria (Villot et al., 2019). In calves supplemented with live yeast *saccharomyces boulardii*, Fomenky et al. (2018) observed an increased bacterial genus such as *Ruminococcaceae* UGG 005, *Roseburia*, and *Olsenella*, which might be related to diarrhea. Villot et al. (2019) analyzed fecal microbiota of unsupplemented and live yeast supplemented calves and observed higher abundance of *Fecalibacterim spp* in supplemented calves. *Fecalibacterium* is known as a butyrate producer, and butyrate may enhance the integrity of the intestinal epithelial barrier (Schwiertz et al., 2010). A higher prevalence of *fecalibacterium* in fecal samples of calves was associated with higher weight gain and lower diarrhea incidence during preweaning period (Oikonomou et al., 2013).

A part of the trial was carried out during summer in Italy which is characterized by heat stress conditions. It is well known that heat stress increases intestinal permeability and the risk of bacterial, viral and parasitic infections (Pearce et al., 2013; Zhai et al., 2019). It is possible that the use of Actisaf® Sc47 PWD in milk replacer exerts a protective action on the intestine. In a study with veal calves, the supplementation of *Saccharomyces cerevisiae* boulardii in milk increased butyric acid-producing bacteria and lactobacillus, along with a reduction of Colinsella, a bacterium previously correlated with increased intestinal permeability (Villot et al., 2019). Actisaf® Sc47 PWD is thought to improve animal performance by maintaining a beneficial intestinal environment, preventing pathogenic bacteria from binding to enterocytes, improving intestinal permeability, and by modulating immune function.

To optimize profitability, individual antibiotic treatment was unavoidable during this trial as it was a commercial farm. Costs associated with treatments were generally lesser for calves supplemented with Actisaf® Sc47 PWD. Feeding cost also decreased for Actisaf® Sc47 PWD groups than CON. The lower feeding cost for calves fed Actisaf® Sc47 PWD was due to better feed efficiency. The use of Actisaf® Sc47 PWD resulted in overall savings of 58.85€. Yan et al. (2005) observed that it cost less to raise calves supplemented with a yeast culture (20 g/d) by up to 29.98 % in a 60-day experiments. In the same way, Magalhães et al. (2008) observed numerical improvement in net income at approximatively $48/calf of calves fed yeast culture than control.

Wither height and body length gain for calves supplemented with Actisaf® Sc47 PWD were significantly greater than that for controls. Our results are consistent with those presented by Lesmeister et al. (2004). Increased structural growth in calves receiving Actisaf® Sc47 PWD may be the result of additional energy and nutrients available for skeletal deposition due to the observed better DMI for supplemented calves. However, this occurrence is not certain because of the limited indication of a yeast culture influence on structural growth, in mature or immature ruminants, in the literature.

## 5 Conclusion

The main objective of farmers is to reduce inputs such us feeds and veterinary treatments and increase performance such us health and growth performance because they can positively affect long term performance. In our study, we demonstrated that daily administration of Actisaf® Sc47 PWD at 1 g per animal via MR improved health status, reduced veterinary treatments and increased growth performance and feed efficiency which validate our hypothesis. Actisaf® Sc47 PWD seems to provide benefits to calves by reducing the incidence and severity of diarrhea. Yeast probiotic Actisaf® Sc47 PWD might play a role in the reduction antibiotics especially in Italy where farm antibiotic use is exceptionally high. We analyzed only zootechnical performances and some health issues. Further studies should be performed to better understand the effect of Actisaf® Sc47 PWD on digestibility, gut microbiota and its effect on immune system and intestinal integrity under normal or challenged conditions.

## Ethical statement

The experimental trial was done in line with the Italian requirements on animal welfare, according to the Legislative Decree n° 126/2011 on calf management. No experimental practices that will or can harm the animals or put their welfare at risk were done.

## Data availability

Research data is confidential. The data/models were not deposited in an official repository. The data/models that support the study findings are available from the authors upon request.

## CRediT authorship contribution statement

**Nizar Salah:** Writing – original draft, Writing – review & editing, Software, Formal analysis, Conceptualization. **Héloïse Legendre:** Writing – review & editing, Validation, Software, Formal analysis. **Valentin Nenov:** Writing – review & editing, Resources, Project administration. **Maxime Briche:** Writing – original draft, Visualization. **Flore Serieys:** Writing – review & editing, Visualization, Resources. **Silvia Grossi:** Writing – review & editing, Resources, Investigation, Data curation, Conceptualization. **Carlo Angelo Sgoifo Rossi:** Writing – review & editing, Supervision, Investigation, Data curation, Conceptualization.

## Declaration of Competing Interest

The authors declare that they have no known competing financial interests or personal relationships that could have appeared to influence the work reported in this paper.
